# THE ASSISTED LIVING SETTING: CLINICAL CARE AND OUTCOMES

**DOI:** 10.1093/geroni/igz038.877

**Published:** 2019-11-08

**Authors:** Barbara Resnick

**Affiliations:** University of Maryland School of Nursing, Baltimore, Maryland, United States

## Abstract

Although the description of assisted living (AL) varies by state this term generally refers to residences that provide housing and supportive services, 24-hour supervision, and at least two meals a day to meet the individual needs of residents. Approximately 37% of residents in AL need help with three or more activities of daily living (ADLs), 42% have some cognitive impairment and 39% need skilled nursing services. Approximately 15 to 50% of older adults living in AL communities experience a fall over a 6 to 24 month period. The cause of these falls involves multiple factors at the resident and facility level. In addition to a high rate of falls there is a high rate of polypharmacy, using the polypharmacy definition of taking five or more medications daily. Polypharmacy results in negative outcomes such as falls and hospitalizations among AL residents. Along with high rates of falls and polypharmacy there is also a high incidence of pain among AL residents and concerns that some pain goes unreported and untreated. Pain, polypharmacy and falls can all influence life satisfaction along with other factors such as the environment itself. The purpose of this symposium is to describe the incidence and factors that influence falls, polypharmacy, pain and pain management and the impact of these care concerns, among others, on life satisfaction drawn from 508 residents from 54 nursing homes participating in the first two cohorts of the study testing the implementation of Function Focused Care for Assisted Living (FFC-AL-EIT).

